# The Cellular Stability Hypothesis: Evidence of Ferroptosis and Accelerated Aging-Associated Diseases as Newly Identified Nutritional Pentadecanoic Acid (C15:0) Deficiency Syndrome

**DOI:** 10.3390/metabo14070355

**Published:** 2024-06-23

**Authors:** Stephanie Venn-Watson

**Affiliations:** 1 Seraphina Therapeutics Inc., San Diego, CA 92106, USA; svennwatson@seraphinatherapeutics.com; 2 Epitracker Inc., San Diego, CA 92106, USA

**Keywords:** ferroptosis, pentadecanoic acid, aging-related diseases, nutritional deficiencies

## Abstract

Ferroptosis is a newly discovered form of cell death caused by the peroxidation of fragile fatty acids in cell membranes, which combines with iron to increase reactive oxygen species and disable mitochondria. Ferroptosis has been linked to aging-related conditions, including type 2 diabetes, cardiovascular disease, and nonalcoholic fatty liver disease (NAFLD). Pentadecanoic acid (C15:0), an odd-chain saturated fat, is an essential fatty acid with the primary roles of stabilizing cell membranes and repairing mitochondrial function. By doing so, C15:0 reverses the underpinnings of ferroptosis. Under the proposed “Cellular Stability Hypothesis”, evidence is provided to show that cell membranes optimally need >0.4% to 0.64% C15:0 to support long-term health and longevity. A pathophysiology of a newly identified nutritional C15:0 deficiency syndrome (“Cellular Fragility Syndrome”) is provided that demonstrates how C15:0 deficiencies (≤0.2% total circulating fatty acids) can increase susceptibilities to ferroptosis, dysmetabolic iron overload syndrome, type 2 diabetes, cardiovascular disease, and NAFLD. Further, evidence is provided that C15:0 supplementation can reverse the described C15:0 deficiency syndrome, including the key components of ferroptosis. Given the declining dietary intake of C15:0, especially among younger generations, there is a need for extensive studies to understand the potential breadth of Cellular Fragility Syndrome across populations.

## 1. Introduction

Based on A.J. Hulbert’s Cell Membrane Pacemaker Theory of Aging, mammalian longevity is determined by the stability of fatty acids in cell membranes, which protect against lipid peroxidation and slow the onset of diseases that eventually lead to mortality [1]. In support of this theory, Hulbert showed that cell membranes containing fatty acids with higher saturation had greater cellular stability. In turn, this stability resulted in lowered circulating lipid peroxidation and was associated with longer mammalian species’ lifespans. There is evidence that this association between cell membrane stability and lifespan is not limited to variations between species but also within species, including humans.

Just over a decade ago, a new form of cell death, called ferroptosis, was discovered [2]. Prior to this discovery, there were only three recognized types of cell death: apoptosis, necrosis, and autophagy. Ferroptosis involves the lipid peroxidation of fragile polyunsaturated fatty acids in cell membranes, which combines with abnormal intracellular iron to induce mass production of reactive oxygen species, resulting in disabled mitochondria and cell death. Ferroptosis has been linked to many aging-related diseases in humans, including type 2 diabetes, cardiovascular disease, NAFLD, and neurodegenerative diseases [3,4,5,6]. Despite over 10,000 published peer-reviewed papers on ferroptosis since its discovery in 2012, it is not clear how or why ferroptosis emerged.

This review introduces the “Cellular Stability Hypothesis”, which proposes that C15:0, an odd-chain saturated fatty acid newly discovered as essential, is integral to stabilizing and strengthening cell membranes. Further, when red blood cell membrane C15:0 concentrations are near or below 0.2% of total fatty acids, the result is ferroptosis. Herein, evidence of the pathophysiology of ferroptosis is provided, which starts with nutritional C15:0 deficiencies and culminates in accelerated metabolic, liver, and cardiovascular disease. This proposed nutritional C15:0 deficiency syndrome (termed “Cellular Fragility Syndrome”) is a form of accelerated cellular aging that may explain why aggressive forms of aging-associated diseases are increasing among younger people. Importantly, this deficiency is fixable.

## 2. Relevance of Ferroptosis to Rising Metabolic and Related Diseases

Over recent decades, there has been an alarming rise in type 2 diabetes, cardiovascular disease, and specific types of related cancers, especially among younger people [7,8,9]. Further, NAFLD emerged in the 1980s and now affects one in three people globally, including one in ten children [10,11]. These chronic cardiometabolic and liver conditions are intricately linked, and there are no clear explanations why these conditions are increasing concurrently and aggressively. As described below, ferroptosis is a shared underlying driver that can explain, at least in part, how type 2 diabetes, cardiovascular disease, and NAFLD have become so intertwined.

### 2.1. Core Features of Ferroptosis: Lipid Peroxidation and Iron Overload

**Figure 1:**
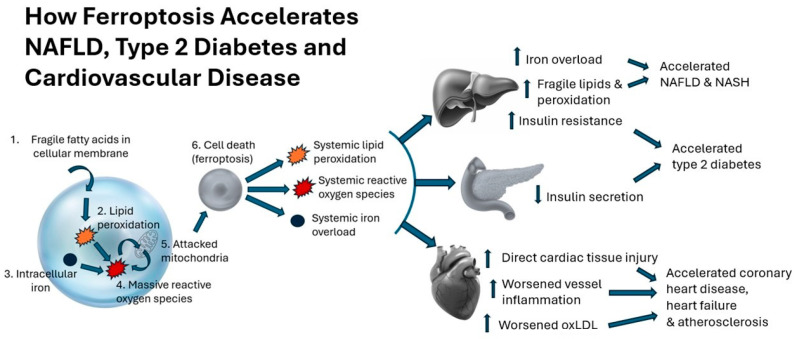
Description of cell death via ferroptosis and its effect of accelerating NAFLD, type 2 diabetes, and cardiovascular disease.

Ferroptosis requires two instigators: fragile polyunsaturated fatty acids in cell membranes that are susceptible to lipid peroxidation and excess intracellular iron [12,13]. Polyunsaturated fatty acids are particularly susceptible to lipid peroxidation because these lipids have multiple double bonds in their molecular structure that are more easily attacked by oxygen [14]. Lipid peroxidation, in and of itself, is a major contributor to numerous aging-related diseases, including metabolic syndrome, NAFLD, cardiovascular disease, and neurodegenerative diseases [15,16,17,18]. Further, dysregulated iron metabolism and direct tissue damage caused by iron overload are being increasingly recognized as drivers of type 2 diabetes, NAFLD, cardiovascular disease, and neurodegenerative diseases [19,20,21,22]. As described below, when lipid peroxidation combines with iron overload, the result is ferroptosis, which has wide-ranging effects that contribute to the onset and progression of metabolic and related diseases (Figure 1).

### 2.2. Type 2 Diabetes

While an estimated 6% of the world’s population currently has type 2 diabetes, this number is expected to increase to 10%, or 1.25 billion people, by 2050 [23]. Even more concerning is the anticipated 673% increase in cases over the next 40 years among people younger than 20 years old [7]. Type 2 diabetes in children is particularly aggressive, with complication and mortality rates that exceed type 1 diabetes [24]. Studies support that ferroptosis is playing a role in the rising incidence of type 2 diabetes, in part due to a one–two punch that contributes to the onset and progression of disease [2]. First, pancreatic cells are more susceptible to ferroptosis relative to other cells due to naturally lower production of a key antioxidative enzyme called glutathione peroxidase; in turn, the ferroptosis of pancreatic cells results in lower insulin secretion [25]. Second, ferroptosis in the liver, another organ susceptible to lipid peroxidation and iron overload, can cause insulin resistance [26]. Together, these effects can impair glucose metabolism and support sustained hyperglycemia. It is well established that people with type 2 diabetes have a higher risk of co-morbidities, including cardiovascular disease, cancer, neurodegenerative diseases, and advanced NAFLD [27,28,29,30].

### 2.3. Nonalcoholic Fatty Liver Disease

NAFLD is a disease involving excessive fat deposition in the liver (i.e., steatosis) that can progress to inflammation, necrosis, and cirrhosis. This advanced stage of NAFLD is called nonalcoholic steatohepatitis (NASH). While the first 20 cases of NAFLD and NASH were reported in 1980, this disease has rapidly increased globally to affect one in three people today [10,11]. NAFLD is the leading cause of hepatocellular carcinoma and soon to become the leading cause of liver transplants. In addition to type 2 diabetes and hepatocellular carcinoma, people with NAFLD also have a higher risk of having cardiovascular disease and non-liver cancers [31,32]. Due to this liver condition’s close ties to metabolic syndrome, the terminology of NAFLD has been changing to metabolic-dysfunction-associated steatotic liver disease (MASLD) [33,34]. Ferroptosis is believed to play an important role in the pathophysiology of NAFLD, especially in relation to the progression of NAFLD to NASH [4,35]. Because the liver is a key organ responsible for lipid and iron metabolism, the vicious cycle of ferroptosis and liver injury may serve as the epicenter for ferroptosis throughout the body.

### 2.4. Cardiovascular Disease

Cardiovascular disease continues to be the leading cause of mortality globally, and the incidence of coronary heart disease has been increasing for adults younger than 45 years old in the U.S. [8,36]. Ferroptosis, due to both lipid peroxidation and iron accumulation in vessel walls, can cause or worsen inflammation and oxidized-LDL-associated atherosclerosis [37]. Ferroptosis has also been linked to coronary heart disease, multiple cardiomyopathies, and heart failure due to direct cardiac tissue injury [5]. Early studies in relevant animal models have demonstrated the potential to prevent or treat cardiovascular diseases by targeting ferroptosis [38].

### 2.5. Neurodegenerative Disease

The incidence of Alzheimer’s disease and Parkinson’s disease has been increasing, primarily due to more people living longer. The brain contains a substantial amount of lipids and, as such, is susceptible to lipid peroxidation. Further, iron deposition in the brain, which increases with age, can cause histologic changes consistent with Alzheimer’s disease [22]. Together, lipid peroxidation and iron accumulation from ferroptosis feed off each other, creating a destructive cycle that contributes to the onset and progression of neurodegenerative diseases [39]. As such, molecules that target ferroptosis have been proposed as potential therapeutics for neurodegenerative diseases [40].

### 2.6. Therapeutic Approaches to Ferroptosis

Understanding that ferroptosis involves lipid peroxidation, intracellular iron, damaging reactive oxygen species, and impaired mitochondria, it makes sense that therapeutic approaches to date have included antioxidants, iron chelators, and mitochondrial protectors [41]. One of the most studied means to reverse ferroptosis is the upregulation of the glutathione peroxidase 4 enzyme (GPX4), which converts damaging lipid peroxides into safer alcohols [42]. These studies, however, are early and limited due to heavy reliance on rodent models and poor understanding of real-life underlying drivers for ferroptosis.

## 3. Essentiality of C15:0, Evidence of Nutritional Deficiencies, and Relevance to Metabolic and Related Diseases

While ferroptosis can increase the risk of type 2 diabetes, cardiovascular disease, and NAFLD, higher circulating concentrations of C15:0 (pentadecanoic acid) have been linked to a decreased risk of these diseases [43,44,45]. This section summarizes the following: (1) evidence of C15:0 as an essential fatty acid and (2) evidence of nutritional C15:0 deficiencies and relevance to type 2 diabetes, cardiovascular disease, and NAFLD.

### 3.1. Evidence of Essentiality

An essential fatty acid is a nutrient required by our bodies to maintain physiological health [46]. Since our bodies are unable to make enough of these fatty acids on their own, we must obtain adequate amounts routinely from our diet. Prior to the discovery of C15:0 as an essential fatty acid, there were only two known essential fatty acids: alpha-linolenic acid (an omega-3 fatty acid) and linoleic acid (an omega-6 fatty acid) [46,47,48]. Evidence of C15:0 as an essential fatty acid includes the following:

1. Dietary C15:0 intake is directly and reliably correlated to circulating C15:0 concentrations, demonstrating that C15:0 is primarily exogenous [49].
2. People with low C15:0 concentrations consistently have a higher risk of developing type 2 diabetes, cardiovascular disease, and NAFLD or NASH [43,44,45].
3. C15:0 supplementation effectively raises circulating C15:0 concentrations and attenuates components of type 2 diabetes, cardiovascular disease, and NAFLD in relevant animal models and humans [47,50,51].
4. C15:0 has dose-dependent mechanisms of action, at concentrations consistent with circulating C15:0 levels, which directly target type 2 diabetes, cardiovascular disease, and NAFLD. These C15:0 mechanisms of action include AMPK and PPARα/δ activation [47,52].

Combined, these studies show that C15:0 is meeting the criteria of an essential fatty acid. Ongoing and future studies will continue to evaluate how well C15:0 meets these criteria.

### 3.2. Evidence of Nutritional C15:0 Deficiencies

As an essential fatty acid, there should be evidence of a nutritional C15:0 deficiency syndrome among people with low C15:0 concentrations. Well-known examples of vitamin deficiencies are vitamin D and rickets, as well as vitamin C and scurvy. In this section, we will review (1) evidence of normal C15:0 concentrations, (2) evidence of low C15:0 concentrations associated with type 2 diabetes, cardiovascular disease, and NAFLD, and (3) drivers of population-wide lower C15:0 concentrations over the past 50 years.

#### 3.2.1. Normal C15:0 Concentrations in Humans

Since C15:0 is often used as a reference fatty acid to measure other fatty acids, C15:0 has been excluded from many population-based studies reporting normal fatty acid concentrations. Population-based studies since the late 1990s that included measurements of C15:0, however, reliably report average C15:0 concentrations at or around 0.2% of total fatty acids [53,54,55]. This is remarkably consistent across age, sex, and geographic location, as well as in plasma, serum, and erythrocyte membrane samples.

Among 1180 adults from seven countries, mean plasma C15:0 concentrations ranged from 0.16% to 0.24% of total fatty acids [53]. Similarly, plasma C15:0 concentrations among healthy adolescents ranged from 0.19% to 0.22% (equivalent to 3.7 to 4.5 µg/mL), based on how much dairy fat was in their diet. This same study also reported erythrocyte membrane C15:0 concentrations that ranged from 0.19% to 0.24% of total fatty acids [54]. Similarly, erythrocyte C15:0 concentrations among healthy men on a high-saturated-fatty-acid diet averaged 0.20%, while those on a high-unsaturated-fatty-acid diet had C15:0 concentrations of 0.17% [55].

When looking at adipose tissue, a 1998 study reported C15:0 concentrations of 0.23% ± 0.09% in women of reproductive fertile age [56]. Interestingly, one of the earliest reports of C15:0 in human samples was from 1962, which showed C15:0 adipose tissue concentrations ranging from 0.4% to 2.8%, which is substantially higher than the studies conducted above during the 1990s through 2020s [57]. Support for health benefits garnered from higher C15:0 concentrations in humans comes from a recent study showing that people living in a High-Longevity Zone (i.e., Blue Zone) have a mean plasma C15:0 concentration of 0.64%, which is significantly higher than people in Low-Longevity Zones (0.29%) [58]. In summary, these studies demonstrate average ranges of circulating C15:0 among healthy people between 0.16% and 0.64%, with the highest levels being in one of the world’s five Blue Longevity Zone populations.

#### 3.2.2. Associations between Lower C15:0 Concentrations and Increased Risk of Type 2 Diabetes, Cardiovascular Disease, and NAFLD

For a long time, due to the relatively low concentrations of C15:0 compared to other fatty acids, C15:0 was assumed to be an inactive and irrelevant fat. It is now well understood that people with lower C15:0 concentrations have a higher risk of developing or having multiple chronic conditions, including type 2 diabetes, cardiovascular disease, and NAFLD.

**Type 2 diabetes.** People with lower circulating C15:0 concentrations are more likely to develop type 2 diabetes over time. In a meta-analysis including 33 prospective cohort studies and 95,810 adults, people with lower C15:0 had a higher risk of developing type 2 diabetes [59]. A second meta-analysis including 16 prospective studies across 12 countries also concluded that lower C15:0 was associated with a higher risk of incident type 2 diabetes [44]. In a third meta-analysis with 19 prospective studies, higher C15:0 had an overall protective effect against type 2 diabetes [43]. Further, a 9-year follow up study including 334 adults with pre-diabetes showed that every 200 g of increased daily intake of high-dairy fat (an approximate equivalent of 200 mg of daily C15:0) increased the odds of achieving normoglycemia by 69% versus progressing to type 2 diabetes [60]. In a more extensive study, a meta-analysis of data from 49 prospective cohort studies showed that people with the highest tertile of circulating C15:0 had a 24% lower risk of developing type 2 diabetes compared to people at the lowest tertile of C15:0 [61].

Similar associations between lower C15:0 and a higher risk of disease have been reported in type 2 diabetes-related conditions, including metabolic syndrome, kidney stones, retinal microangiopathy, and cognitive decline. Japanese men with metabolic syndrome had lower serum phospholipid C15:0 concentrations compared to healthy controls (0.15% vs. 0.17%) [62]. A study evaluating C15:0 concentrations between people with or without kidney stones showed that for every unit increase in serum C15:0 concentration, the risk of kidney stones was reduced by 2%; this risk decreased even more, to 5% for every unit increase in C15:0, among people with type 2 diabetes [63]. In another study with 166 adults at risk of type 2 diabetes, higher serum C15:0 concentrations were predictive of a lower risk of developing retinal microangiopathy (C15:0 concentrations of cases = 0.26%, healthy controls = 0.28%) [64]. Further, among adults with type 2 diabetes, those with higher plasma C15:0 had better cognitive function, which included higher total Mini-Mental State Examination (MMSE) scores and MMSE delayed recall scores, as well as better total Montreal Cognitive Assessment (MoCA) and MoCA visual–spatial ability scores [65].

**Cardiovascular disease.** Numerous studies have repeatedly shown that people with lower C15:0 concentrations have a higher risk of developing cardiovascular disease and related conditions. In a meta-analysis including 18 cohort studies, lower circulating C15:0 concentration was associated with a higher risk of cardiovascular disease [66]. Further, in this study, there was a linear correlation between higher serum cholesterol ester C15:0 concentrations and lower cardiovascular disease risk, with protective effects starting at around 0.2% and gaining protective benefits through the highest measurements of 0.55%. In a separate study, among 45- to 75-year-olds, lower adipose C15:0 concentrations (0.37%) were associated with a higher risk of first myocardial infarction compared to healthy controls (0.41%) [67]. This finding was repeated in a prospective case–control study with 234 participants, where cases who developed a first myocardial infarction had lower serum phospholipid C15:0 concentrations compared to healthy controls (0.21% vs. 0.22%) [68].

Further, the study above showed that combined serum C15:0 + C17:0 phospholipid concentrations were inversely correlated with triglycerides, cholesterol, insulin, leptin, and PAI-1. This finding is consistent with a large prospective cohort study, which included 15,919 adults and showed that plasma phospholipid C15:0 was negatively correlated with total cholesterol and triglycerides [45]. In an older population including 2140 people having a mean age of 77 years who were followed for a median of 9.7 years, those with lower serum C15:0 concentrations were more likely to develop heart failure [69]. Correlations between higher circulating C15:0 concentrations and lower risk of heart failure were repeated in a meta-analysis including 13 prospective cohort studies with 7680 cardiovascular disease cases [70]. The associated protective effect of C15:0 to the heart extends to infants; using untargeted metabolomics on maternal amniotic fluid to identify molecules related to the severity of congenital heart disease, mothers with higher C15:0 concentrations were less likely to have infants with moderate (vs. mild) congenital heart disease [71].

**NAFLD.** Similar to type 2 diabetes and cardiovascular disease, lower circulating C15:0 concentrations are correlated with an increased risk of NAFLD. Concentrations of C15:0 in erythrocytes are positively correlated with C15:0 in the liver, and a large prospective cohort study with 15,919 adults showed that plasma phospholipid C15:0 concentrations are inversely correlated with liver enzymes ALT, AST, and GGT [45,72]. Beyond liver enzymes, adults with lower plasma free fatty acid C15:0 concentrations have higher liver fat and higher glucose levels; this study also showed that healthy controls consumed more total dairy products (average of 4.4 servings a day) and butter (average of 1.9 servings a day) compared to people with NAFLD, who ate 2.8 and 0.8 servings a day of total dairy product and butter, respectively [73]. Similarly, among 237 children aged between 8 and 17 years old, higher plasma C15:0 concentrations predicted lower liver fat, as did eating more daily dairy fat [74]. These studies show that low circulating C15:0 is associated with higher liver enzymes and NAFLD.

The culmination of studies in this section demonstrates that lower circulating C15:0 is correlated with an increased risk of type 2 diabetes, cardiovascular disease, and NAFLD in children and adults. Surprisingly, there appears to be little tolerance for even slight decreases around or below 0.2% C15:0 concentrations, which have been repeatedly associated with a higher risk for various chronic diseases. As shared below, this pattern is also evident during our earliest periods of development.

#### 3.2.3. Importance of Normal C15:0 Concentrations to Support Healthy Pregnancies and Early Child Development

As an essential fatty acid, there should be evidence that C15:0 is an important nutrient not only to support the health of children and adults but of infants, too. The first testament to C15:0’s role in protecting infant health is the presence of C15:0 in human colostrum and milk. C15:0 content in human colostrum varies from 0.04% to 0.58% of total fatty acids, with the lowest concentrations reported in women from Poland and the highest in women in Australia [75]. Even higher C15:0 concentrations, ranging from 0.61% to 0.89%, have been reported in colostrum from women in Slovenia [76]. Relatedly, among a wide variety of animal meat products listed in the United States Department of Agriculture FoodData Central, Australian lamb and Australian grass-fed beef are the top two highest C15:0-containing meats, with 0.47% and 0.36% C15:0, respectively [77]. More studies are needed to better understand how regional and cultural diets influence dietary C15:0 intake and resulting C15:0 concentrations in circulation, as well as in mothers’ colostrum and milk.

Beyond colostrum, C15:0 content in human milk varies from 0.12% to 0.39% of total fatty acids, with the lowest concentrations reported in women from China and highest among women in Australia [78]. Aligned with these ranges, human milk from women in Taiwan had maintained average C15:0 concentrations of around 0.2% throughout 297 days of lactation [79]. These findings are aligned with cultures that drink less animal milks (China and Taiwan) compared to those that consume dairy products, including milkfat from grass-fed cows and cattle. Similarly, another study showed that mean C15:0 content in human milk was 0.38%, while formula milks contained only 0.004% C15:0. Given these results, the authors of this study suggest that formula milks may need to be supplemented with C15:0 to better match human milk [80].

Paralleling extensive studies linking low circulating C15:0 concentrations to poorer health in adults, low C15:0 has also been associated with higher risk pregnancies, poorer infant development, and poorer cognition among children. Specifically, women with lower mean plasma phospholipid C15:0 levels during gestational weeks 10–14 (cases, 0.23% vs. controls, 0.24%) and gestational weeks 15–26 (cases, 0.24% vs. controls, 0.25%) were more likely to develop gestational diabetes mellitus compared to healthy pregnancy controls [81]. Beyond pregnancies, 18 mother–infant pairs were prospectively followed over the first 6 months of exclusive breastfeeding, and fatty acids were measured in the mothers’ milk, along with the infants’ daily intake. This study showed that a lower daily infant intake of C15:0 was correlated with smaller head circumference, less healthy body weight, and less healthy BMI [82]. Another prospective study following 1200 mother–child pairs over 6 years showed that mothers who exclusively breastfed for 6 months and had higher C15:0 concentrations in their erythrocyte membranes and colostrum had children who had higher language development and overall better cognition at age 3, as well as better verbal IQ scores at ages 5 to 6 years old [83].

In summary, the studies above support that minor changes in circulating C15:0 are associated with meaningful and clinically relevant impacts to our metabolic, cardiovascular, and liver health as adults, as well as our growth and development as children and infants. Identifying optimal diets to raise and maintain C15:0 concentrations, which may include mimicking the diets of people living in Sardinia’s High-Longevity Zone who have the highest circulating C15:0 concentrations (0.64% of total fatty acids), will be important to help individuals make the best dietary choices to support their long-term health [58]. Interestingly, the Sardinian diet is heavily dairy-based, with up to 26% of their diet being dairy products, primarily from local mountainous grass-fed sheep and goat’s milk that have relatively high C15:0 content [84]. The next section will review drivers of declining dietary C15:0 intake over the past half-century, which has resulted in declining circulating C15:0 and coinciding susceptibilities to type 2 diabetes, cardiovascular disease, and NAFLD.

#### 3.2.4. Drivers for Declining C15:0 Concentrations

A prospective study following 722 adults over 13 years showed a significant decline in mean plasma phospholipid C15:0 concentrations; specifically, mean population C15:0 levels were 0.23% during 1993–1997, 0.21% during 1998–2000, and 0.20% during 2004–2011 [85]. There are at least three primary drivers for declining C15:0: (1) lower dietary intake of foods containing C15:0, (2) lower C15:0 content in foods due to changing environments and practices, and (3) natural declines in circulating C15:0 concentrations that occur with age.

**Lower dietary intake of whole-fat cow’s milk.** Given that circulating C15:0 has long been reliably and routinely used as a biomarker of dairy fat intake, it is well established that milkfat is the primary dietary source of C15:0, especially in Western countries [86,87]. For example, eating more butter, full-fat dairy products, and whole-fat milk is positively correlated with higher plasma phospholipid C15:0 concentrations [88]. In a cohort study involving over 2000 pregnant women, those who ate more cheese had higher erythrocyte, umbilical cord erythrocyte, and colostrum C15:0 concentrations. Conversely, women who ate more reduced-fat dairy products had lower erythrocyte and colostrum C15:0 concentrations [89]. These studies show that full-fat dairy products (versus reduced-fat products) are needed to significantly raise circulating C15:0 levels.

In 1977, the U.S. Congress released dietary recommendations for all Americans, which emphasized the need to reduce the intake of all dietary saturated fatty acids, especially by avoiding whole dairy fat, including butter and whole-fat milk [90]. These recommendations continue to be included in current USDA, American Diabetes Association, American Academy of Pediatrics, American Heart Association, and the World Health Organization dietary guidelines [91,92,93,94,95,96,97,98,99,100].

As a direct result of these recommendations, there have been dramatic population-wide declines in whole-fat dairy milk consumption. A 2022 USDA report titled “Fluid Milk Consumption Continues Downward Trend, Proving Difficult to Reverse” shared that the amount of daily cow’s milk (including nonfat, low fat, and whole fat) ingested by the average adult in the U.S. declined from two cups during the 1950s, to one cup in the 1970s, to half a cup by 2019. Milk consumption dropped at the highest rate during the 2010s compared to the previous six decades [96]. Given an approximate 100 mg of C15:0 per cup of whole milk, these trends in milk consumption translate to a decline from 200 mg to 50 mg of C15:0 a day over the past 70 years [77]. Based on pharmacokinetic data, in which every 100 mg of C15:0 ingested results in an approximate 10 µM increase in circulating C15:0, circulating C15:0 levels would decrease from 20 µM to 5 µM (an approximate decline from 0.2% to 0.05% of total fatty acids) [97,98].

The decline in cow’s milk consumption has been even more extreme in infants. While almost 100% of infants were provided cow’s milk within their first year of life during the early 1970s, only 10% of infants less than 1 year old had cow’s milk in 1998 [99]. With each new generation, the avoidance of all cow’s milk has increased dramatically, as people are choosing plant-based milks over cow’s milk. Notably, plant-based milks contain negligible C15:0 [100]. Due to the falling demand for cow’s milk, it has been estimated that, by 2030, the number of cows in the U.S. will decline by 50% [101].

Individuals’ C15:0 concentrations readily respond to dietary C15:0 changes; 19 days after moving from a diet high in saturated fatty acids (18% total energy) to a diet high in polyunsaturated fatty acids, healthy adult plasma phospholipid C15:0 concentrations decreased significantly, from 0.35% to 0.27%, and erythrocyte membrane C15:0 concentrations decreased from 0.51% to 0.38% [102]. Similarly, when healthy adolescents ate ≥4 servings of dairy products a day, their erythrocyte C15:0 concentrations increased over 12 months [54]. Combined, these studies demonstrate that population-wide milkfat consumption is rapidly declining, and that these declines will be difficult to reverse. In turn, the lowered intake of milkfat lowers circulating C15:0 levels, setting the stage for population-wide nutritional C15:0 deficiencies. Beyond decreasing dietary intake of dairy products, C15:0 levels may be declining due to changes in C15:0 content within milkfat.

**Lower C15:0 content in whole-fat cow’s milk.** In addition to population-wide declines in milkfat intake, differences in cows’ breed, milking season, and feed type can significantly affect the amount of C15:0 in whole milkfat [103]. Thus, even among people eating or drinking products with whole milkfat, there may be lower C15:0 content than expected. For example, there is a positive linear relationship between the percentage of grass (versus corn silage) fed to cows and C15:0 content in their milk; cows fed 0%, 30%, 60%, and 100% fresh grass (conversely, 100%, 70%, 40%, and 0% corn silage) had milkfat containing 0.77%, 0.99%, 1.1%, and 1.3% C15:0, respectively. This translates to nearly a 2-fold difference in dietary C15:0 intake, even from whole milkfat, depending on what cows are fed [104]. Even among fresh-grass-eating cows, there are differences in C15:0 milkfat content based on the season and types of grass fed. For example, C15:0 milkfat content from cows grazing in the spring was 1.21% total fatty acids, while the same grazing cows produced milk with 0.96% C15:0 in the winter [105]. Further, cows grazing on *Trifolium alpinum* pastures have more C15:0 in their milkfat than cows grazing on *Festuca nigrescens* [106]. Given these differences, there is a need to evaluate how changes in cows’ breed, feed, and season, which directly affect milkfat C15:0 content, may be contributing to (or could help to address) lower circulating C15:0 concentrations in humans. While dairy fat is the primary dietary source for C15:0 in Western countries, the likely primary source for C15:0 in many Eastern countries is fish.

**Lower dietary intake of fish.** While the declining dietary intake of cow milkfat can explain declining circulating C15:0 concentrations in Western countries, a similar phenomenon of declining C15:0 levels may be occurring in island and Eastern countries due to changing habits related to dietary fish. As seen in Table 1, C15:0 content varies greatly by fish species, with mullet and catfish having C15:0 content similar to milkfat (around 1%) [107]. As such, changes in either the type or the amount of fish eaten may influence one’s circulating C15:0 concentrations.

A longitudinal cohort study measured dietary changes from 1991 to 2009 among two groups in China: those who followed a traditional southern dietary pattern and those who followed a modern high-wheat dietary pattern [108]. Of those following the traditional southern dietary pattern, the percentage of people who routinely ate fish or seafood increased from 34% to 43% over the 18-year study period; this group did not drink animal-based milk. In contrast, of those following the modern high-wheat dietary pattern, the percentage of people who routinely ate fish or seafood decreased from 44% in 1991 to 24% in 2009. While the modern high-wheat dietary group included people who drank animal-based milk, that percentage of people decreased from 64% to 49%. Over the study’s 18-year period, the popularity of the modern high-wheat dietary pattern increased, supporting that more populations in China are eating less fish and seafood. Further studies are needed to evaluate whether these changes, taking into account dairy food intake, have negatively impacted circulating C15:0 levels over time.

Perhaps more important are the global changes in fish that could lower their C15:0 content. When evaluating the body sizes of fish from 1960 to 2020, Martins et al. found that the mean body sizes of fish have been decreasing [109]. This movement toward more smaller fish in the oceans, which may be due to combinations of overfishing and warmer temperature waters, are expected to result in fish that contain less fat, including C15:0. This point is furthered by lower circulating C15:0 concentrations that were observed in dolphins fed lower fat fish, which will be discussed in Section 4. Aside from population-wide declines in the dietary intake of foods containing C15:0 and the effects of changing practices and environments on C15:0 content in dairy fat and fish, there is a third contributor to low C15:0 levels in humans: aging.

**More older populations.** Aging populations are increasing globally, and circulating C15:0 concentrations naturally decline with age. The previously mentioned Sardinia study compared plasma fatty acid profiles among (1) people 65 to 70 years old living in a High-Longevity Zone (HLZ); (2) people > 80 years old living in an HLZ; and (3) people aged >80 years living in a Low-Longevity Zone (LLZ) [58]. This study found that 65- to 70-year-olds living in the HLZ had the highest C15:0 levels (0.64% of total fatty acids), followed by people 80 years or older in the HLZ (0.42%), and last by people 80 years or older in the LLZ (0.29%). This study supports that C15:0 concentrations naturally decline with age and, as shared earlier, that people living in HLZs have substantially higher circulating C15:0 compared to “normal” C15:0 concentrations reported in other population studies shared in Section 3.2.1 (around 0.2%). A separate study assessed the causes of ocular health declines with age, specifically the decreasing integrity of the stratum corneum. When comparing the fatty acid content of the stratum corneum of 110 people over 60 years old with 110 young and healthy participants (10 to 40 years old), C15:0 decreased with age [110]. Given these studies, there is a need to understand why C15:0 declines with age and how declining C15:0 levels may accelerate aging as we become older.

In summary, evidence was provided in this section that supports the following: (1) C15:0 is an essential fatty acid of which adequate concentrations are needed to maintain baseline physiological health; (2) low circulating C15:0 concentrations (around or below 0.2% of total fatty acids) are associated with increased susceptibilities to type 2 diabetes, cardiovascular disease, and NAFLD; and (3) the core drivers of observed population-wide lower C15:0 levels include lower whole milkfat dietary intake, variations in C15:0 in milkfat and fish due to industry and environmental drivers, and natural declines in C15:0 with aging.

While epidemiological studies are helping to determine normal versus abnormal circulating C15:0 levels that are associated with risks of developing type 2 diabetes, cardiovascular disease, and NAFLD, they do not prove causality. Surprisingly, a population of bottlenose dolphins has helped to elucidate the pathophysiology of C15:0-nutritional-deficiency-driven metabolic, cardiovascular, and liver diseases. In turn, this pathophysiologic mechanism brings us closer to a core argument of the Cellular Stability Hypothesis: that C15:0 deficiencies cause ferroptosis and accelerate the onset of downstream aging-related diseases.

## 4. Metabolic Diseases and Iron Overload in Dolphins: Insights on an Emerging C15:0 Nutritional Deficiency Syndrome

### 4.1. Physiologic Similarities between Dolphins and Humans

While the section above provides evidence that lower circulating C15:0 levels are associated with a higher risk of type 2 diabetes, cardiovascular disease, and NAFLD, a pathophysiologic mechanism is needed to justify the proposal of C15:0 nutritional deficiencies. This section reviews learnings from long-lived, large-brained dolphins, which have revealed two central tenets of a proposed C15:0 nutritional deficiency syndrome: increased cellular fragility and iron overload.

Bottlenose dolphins are mammals that once lived on land but re-entered the ocean an estimated 50 million years ago [111]. As deer-like artiodactyls, the land-based predecessor to dolphins was likely primarily a plant eater that evolved to eating fish at sea. We previously proposed that dolphins developed an observed insulin-resistant state to support all-meat diets that require liver production of glucose to meet high demands by their large brains [112]. This hypothesis parallels the same proposed “carnivore connection” in humans, where insulin resistance was an advantageous condition during the Ice Ages, as humans moved from a mixed plant-and-meat diet to pure-meat diets [113].

Further evidence of special parallels between dolphins and humans includes shared rapid glucose GLUT-1 transport in red blood cells as adults; high encephalization quotients (brain-to-body mass ratios); and remarkable synteny of chromosome 1, which has been conserved among only humans, dolphins, great apes, and two-toed sloths [114,115,116]. Thus, dolphins and humans have co-evolved similar mechanisms and metabolism, likely to support glucose demands for large brains and long lives. This may also explain why aging dolphins and humans share similar chronic conditions [117].

The U.S. Navy has cared for a sustained population of approximately 100 bottlenose dolphins, currently living in San Diego Bay, for over 60 years. While dolphins in the wild live an average of 20 years, U.S. Navy dolphins routinely live into their 40s and even 50s [118]. These older dolphins have provided invaluable insight into chronic diseases of aging, including insulin resistance, metabolic syndrome, nonalcoholic fatty liver disease, dysmetabolic iron overload syndrome, and anemia. These insights are provided below.

### 4.2. Fatty Liver Disease, Iron Overload, and Metabolic Syndrome in Dolphins: Dysmetabolic Iron Overload Syndrome

Bottlenose dolphins are naturally susceptible to fatty liver disease that mimics NAFLD in humans, including the presence of steatosis, iron deposition within Kupffer cells (liver macrophages or reticuloendothelial cells), and inflammation [7]. Dolphins with fatty liver disease have phasic increases in liver enzymes (ALT, AST, and/or GGT), and compared to healthy controls, individuals with elevated liver enzymes have higher iron, serum globulins, bilirubin, cholesterol, triglycerides, glucose levels, and erythrocyte sedimentation rate and lower platelet counts [119]. This presentation, especially preferential iron deposition within Kupffer cells, is consistent with iron overload related to aggressive NAFLD in people [120].

In addition to high serum iron (>300 µg/dl) and visualized iron deposition in liver Kupffer cells (hemochromatosis), dolphins with iron overload have hyperferritinemia and moderately elevated transferrin saturation that increase with age [121]. Similar to humans with liver iron overload, the use of routine phlebotomy treatments to reduce body iron stores temporarily lowers serum ferritin, iron, total iron binding capacity, and liver enzyme levels [122]. Ferritin, a measurement of iron storage in the body, is also higher in people with NALFD compared to healthy controls [123].

Naturally occurring metabolic syndrome in dolphins also parallels that in humans. In addition to fatty liver disease and liver iron overload, dolphins with metabolic syndrome have elevated insulin (13 ± 13 µIU/mL), glucose (108 ± 12 mg/dl), triglycerides (128 ± 45 mg/dl), and total cholesterol (217 ± 51 mg/dl) [124]. Those with the highest insulin (>14 µIU/mL) have higher glucose, iron, and GGT compared to healthy controls. While dolphins with metabolic syndrome do not have a higher body mass index (BMI) compared to healthy controls, those with the highest insulin (i.e., greatest insulin resistance) do have a higher BMI compared to controls. These studies show that dolphins have a consistent phenotype of fatty liver disease that includes macrophage-preferential liver iron overload and metabolic syndrome. This presentation in dolphins is remarkably consistent with dysmetabolic iron overload syndrome in humans [125].

### 4.3. Dysmetabolic Iron Overload Syndrome in Humans

Up until 25 years ago, iron overload (also called hemochromatosis) in humans was primarily considered a hereditary disease caused by *HFE* gene mutations that increased gut absorption of dietary iron [126]. Between 1998 and 2000, however, a new, non-hereditary form of iron overload was recognized and referred to as “dysmetabolic-associated liver iron overload syndrome”, “dysmetabolic hepatosiderosis”, or “dysmetabolic iron overload syndrome” (DIOS) [127,128,129]. In 2003, 15% of patients with iron overload had DIOS [130]. Key differentiators between DIOS versus hereditary hemochromatosis include iron deposition in mesenchymal cells (Kupffer cells or other macrophages), presence of metabolic syndrome, and hyperferritinemia with normal transferrin saturation [131,132]. Of the 23 people with DIOS in the 2003 study, 50% had NAFLD or NASH, and 12% had advanced, bridging fibrosis or cirrhosis.

By 2011, DIOS was characterized as “a frequent finding in the general population” and present in approximately 33% of people with NAFLD and metabolic syndrome [133]. Further, DIOS has been identified as a causal factor for more severe NASH, cardiovascular disease, insulin resistance, hyperglycemia, type 2 diabetes, and cancer [133,134,135]. As such, treatments targeting DIOS have the potential to attenuate all these related conditions. People with DIOS not only have higher liver iron levels but also have overall higher mobilized iron and overall body iron stores [136]. Further, DIOS is characterized as a condition involving poor export of iron out of cells, resulting in more cells with intracellular iron [137]. This lays the groundwork for DIOS as the potential epicenter of ferroptosis throughout the body [3].

Interestingly, obesity and NAFLD are associated with both DIOS and iron deficiency, which on the surface appear to be opposite conditions [138]. As shared by Malesza et al., however, both DIOS and iron deficiency involve higher circulating hepcidin and lower ferroportin, suggesting they are different manifestations of a similar problem [136]. This is why the treatment of iron deficiency anemia with iron supplementation can be problematic and why alternative options are actively being sought to address iron imbalance conditions.

Given that the underlying causes of both DIOS and ferroptosis have remained a mystery and that both conditions involve abnormal intracellular iron deposition which emerged during a similar timeframe, it is logical to believe that DIOS and ferroptosis have shared etiologies. Returning to the dolphin studies, a key risk factor for DIOS was discovered: lower dietary and circulating concentrations of odd-chain saturated fatty acids, including C15:0.

### 4.4. Lower Dietary and Circulating C15:0 Are Associated with DIOS in Dolphins

Unlike humans, who have complicated diets, dolphins primarily eat fish. As such, many proposed risk factors for poor health in the human diet (sugar, artificial trans-fatty acids, and ultra-processed foods) can be immediately eliminated as contributors to DIOS, NAFLD, and metabolic syndrome in dolphins. To evaluate potential dietary risk and protective factors for these conditions in dolphins, an initial study used a standard fatty acid panel to measure total fatty acids in dolphins’ archived serum, as well as fish in their diets. This study found that dolphins with higher serum concentrations of OCFAs (C15:0 and C17:0) had lower, healthier insulin levels (2–5 µIU/mL) compared to dolphins with lower C15:0 and C17:0 (insulin = 11 ± 12 µIU/mL) [139]. When different dietary fish types were tested for fatty acids, there was a surprising variation in OCFAs; for example, C17:0 content ranged from no detectable C17:0 in capelin to 164 mg per 100 g in mullet. Thus, there was the opportunity to provide dolphins with more fish containing higher amounts of OCFAs to effectively raise their OCFA levels.

### 4.5. An Unexpected Clue: Increased Dietary C15:0 Alleviates Anemia in Dolphins

As a next step, six dolphins were provided a modified diet containing fish with higher amounts of C15:0 and C17:0. Specifically, the dolphins’ dietary C15:0 intake increased from 1 g to 5 g per day. This modified fish diet resulted in significant linear declines in ferritin over 18 weeks from a mean of 373 ng/mL at Week 0 to 243 ng/mL at Week 18 [139]. Additionally, triglycerides, glucose, and insulin levels normalized among dolphins with abnormal values at the beginning of the study. This study, while limited, suggested that lower circulating OCFAs may not only be associated with a higher risk of DIOS, metabolic syndrome, and NAFLD in dolphins but that increasing dietary OCFA intake may help attenuate these conditions.

Following this pilot study, a larger modified diet study was conducted with 20 dolphins on the modified diet and 10 dolphins on the baseline control diet. The modified fish diet increased daily dietary C15:0 intake from 1.3 g to 4.5 g per day, which resulted in mean serum C15:0 concentration increases from 7 to 28 µg/mL (from 0.27% to 1.2% of total fatty acids) and mean erythrocyte membrane C15:0 concentrations from 1.5 to 5.8 µg/mL (from 0.17% to 0.58% of total fatty acids) by the first month [140]. In addition to dolphins on the modified diet having lower insulin and lower cholesterol by Week 6 compared to control dolphins, the case dolphins also had the unexpected benefit of alleviated anemia.

Among 20 dolphins in the modified diet group, 10 had chronic anemia (hemoglobin < 12.5 g/dl) at the beginning of the study. Of these, anemia resolved in all dolphins by Month 3, which was sustained throughout the 6-month study. Resolved anemia included not only raised hemoglobin but raised hematocrit, total red blood cells, and nucleated (new) red blood cells, as well as lowered red blood cell distribution width (RDW, from 15% ± 2% to 13% ± 1% by Month 6) [140]. Further, using stepwise regression, increased erythrocyte C15:0 cell membrane concentrations from 1.5 to 5.8 µg/mL independently predicted the observed alleviated anemia. This study supported the following: (1) that at baseline, some dolphins had a regenerative anemia with premature red blood cell loss, leading to higher anisocytosis (i.e., RDW), which (2) was alleviated by increasing C15:0 concentrations in red blood cell membranes to 5.8 µg/mL (0.58% total fatty acids). Given this clue of C15:0’s near-term positive effect on anemia and red blood cell indices among dolphins with DIOS, NAFLD, and metabolic syndrome, an appropriate model was sought to test the efficacy of pure C15:0 supplementation on this disease phenotype.

### 4.6. C15:0 Attenuation of Anemia, DIOS, NASH, and Metabolic Syndrome in a Relevant Model

The pathophysiology of fragile red blood cells and anemia leading to iron overload, in association with DIOS, NAFLD, and metabolic syndrome, had been previously elucidated in a Japanese rabbit model that closely mimics these conditions in dolphins and humans [141]. When provided a high-cholesterol, high-fat diet, this model develops insulin resistance, metabolic syndrome, and NASH with bridging fibrosis, as well as anemia due to fragile and oxidized red blood cells, which are engulfed by Kupffer cells, resulting in DIOS and advanced NASH.

In this model, a group treated with daily oral C15:0 (>98.5% free fatty acid C15:0) for 11 weeks had lower total cholesterol, triglycerides, globulins, platelets, and bilirubin; less liver fibrosis (lower risk of advancing to bridging fibrosis) and liver iron deposition; lower nucleated red blood cells, RDW, and reticulocytes; and higher hemoglobin, hematocrit, and red blood cells compared to the non-treated disease group [47]. In summary, C15:0 effectively attenuated key components of this disease phenotype, including fragile red blood cells, DIOS, dyslipidemia, and NASH.

Beyond treating the full suite of our proposed underlying pathophysiology of what causes ferroptosis, C15:0 also attenuates the individual components of ferroptosis. This includes lowering lipid peroxidation, lowering reactive oxygen species, and repairing mitochondria. In a mouse model of NASH, C15:0-treated individuals had significantly lower liver lipid peroxidation compared to the non-treated disease group [50]. Regarding mitochondrial function, our own studies showed that C15:0 repairs mitochondrial function, including a significant reduction in mitochondrial reactive oxygen species; effective C15:0 concentrations were between 10 and 50 µM, with optimal efficacy at 20 µM (5 µg/mL) [47]. This optimal concentration of 20 µM (5 µg/mL) matches that of a robust cell-based activity assessment of C15:0. This emerging critical concentration was also seen in our previously reported dolphin studies, in which the higher-C15:0-content diet resulted in raised erythrocyte membrane C15:0 concentrations from 1.5 to 5.8 µg/mL (from 0.17% to 0.58% of total fatty acids); in turn, crossing over the 0.2% and 5 µg/mL thresholds resulted in stabilized red blood cells and alleviated anemia [140].

This section demonstrates that, beyond just associations between lower circulating C15:0 and a higher risk of multiple chronic diseases, the dolphin model presents the pathophysiology behind how low C15:0 levels in red blood cell membranes (<0.2% of fatty acids) cause ferroptosis and outfall conditions, which start at the liver. Further, this section showed that increasing dietary C15:0 directly increases red blood cell membrane C15:0 levels and that C15:0 directly ameliorated the suite of the proposed pathophysiology behind ferroptosis. This included the ability to use C15:0 to restore red blood cell stability, lower liver iron overload and lipid peroxidation, repair mitochondria and lower reactive oxygen species, and attenuate liver fibrosis and hyperlipidemia.

## 5. Proposed Nutritional-C15:0-Deficiency-Driven Cellular Fragility Syndrome

### 5.1. Proposed Nutritional C15:0 Deficiency Definition

Given the studies above, paired with robust epidemiological studies and well-defined mechanisms of action around C15:0, the following categories are proposed:

- **C15:0 concentrations > 0.2% of total fatty acids are required in cell membranes to ensure cellular stability.** Under this concentration, cell membranes can become fragile and are susceptible to lipid peroxidation and premature death [47,50,140,142].
- **Optimally, circulating C15:0 concentrations should be >0.4% to 0.64%,** which studies show support long-term red blood cell stability, cardiovascular health, and longevity [58,66,140].
- **C15:0 deficiency, defined as ≤0.2% of total circulating fatty acids, results in Cellular Fragility Syndrome**, which includes higher risks of developing DIOS, ferroptosis, anemia, advanced NAFLD and NASH, cardiovascular disease, and type 2 diabetes. In addition to low C15:0 concentrations and indices associated with liver and cardiometabolic diseases, people with Cellular Fragility Syndrome are expected to have (1) hyperferritinemia, (2) elevated lipid peroxidation levels, (3) red blood cells with elevated osmotic fragility, and (4) elevated red blood cell distribution width (RDW) [3,4,5,6,47,140,142].

### 5.2. Proposed Pathophysiology of Nutritional C15:0 Deficiencies (Cellular Fragility Syndrome)

**Figure 2:**
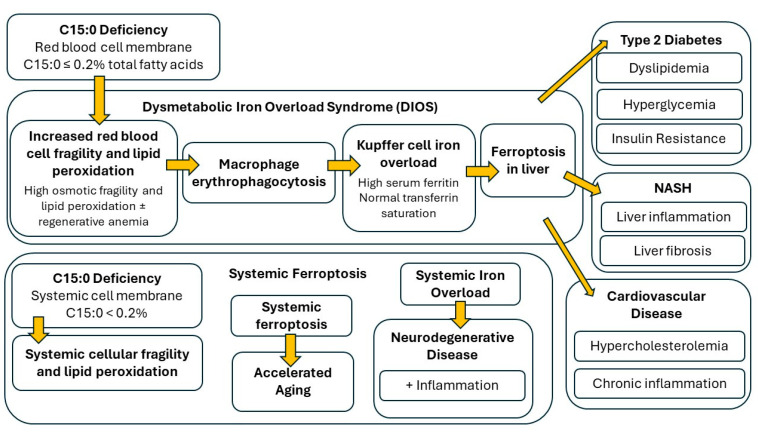
Proposed C15:0 deficiency syndrome (Cellular Fragility Syndrome).

Given the dolphin modified diet and controlled intervention studies, paired with human epidemiological studies and known C15:0 mechanisms of action, the proposed pathophysiology of a nutritional C15:0 deficiency (Cellular Fragility Syndrome) is summarized in Figure 2. The stages are as follows:

- **First, red blood cell membranes containing C15:0 ≤ 0.2% total fatty acids become fragile and susceptible to lipid peroxidation.** In addition to C15:0 measurements, tests to detect this stage may include osmotic fragility tests, RDW, and systemic lipid peroxidation.
- **Second, fragile red blood cells are engulfed by macrophages**, including liver Kupffer cells, which over time results in regenerative anemia and DIOS. Tests that may detect this stage include low hemoglobin, high reticulocytes, high RDW, and hyperferritinemia.
- **Third, combined elevated lipid peroxidation with iron overload results in ferroptosis** in the liver and the subsequent advancement of NAFLD and NASH, including increased inflammation, cell damage, and fibrosis in the liver. Tests that may detect this stage include elevated liver enzymes and increased inflammatory markers (elevated globulins, IL-6, TNFα, and MCP-1).
- **Fourth, impaired liver function and ferroptosis results in insulin resistance, metabolic syndrome, type 2 diabetes, and cardiovascular disease.** Tests to detect this stage include non-specific markers of these diseases, including elevated insulin, glucose, cholesterol, and triglycerides.
- **Finally, spillover iron and systemically fragile cell membranes result in systemic iron overload and ferroptosis**, which pairs with fragile cells to further impair tissues, resulting in accelerated aging, including accelerated cardiovascular disease.

## 6. Demonstrated In Vivo Efficacies of Oral C15:0 Supplementation to Reverse Cellular Fragility Syndrome

**Figure 3:**
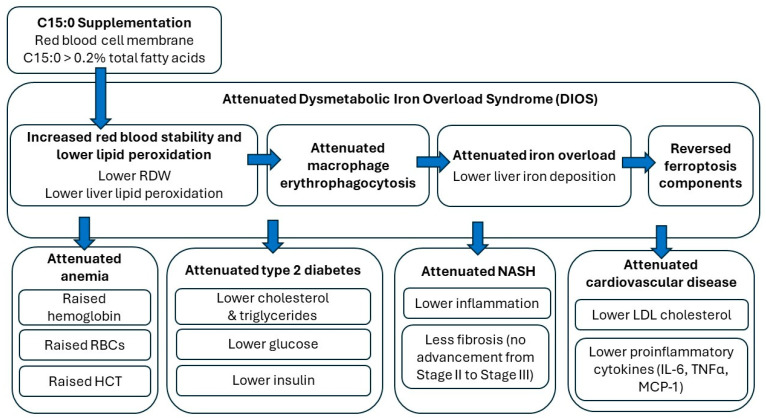
Demonstrated in vivo efficacies of C15:0 dietary supplementation, including reversal of Cellular Fragility Syndrome.

If the proposed Cellular Fragility Syndrome is caused by nutritional C15:0 deficiencies, there should not only be evidence that low C15:0 levels cause this syndrome but that it can be reversed with C15:0 supplementation. As described in Figure 3 and below, controlled studies have shown that C15:0 supplementation reverses key components of Cellular Fragility Syndrome.

### 6.1. C15:0 Supplementation Stabilizes Red Blood Cells, Attenuates Anemia, and Lowers Lipid Peroxidation

Anemia was attenuated within 1 to 3 months in a model of anemia, DIOS, and NASH supplemented with C15:0, with effects including raised hemoglobin and lowered reticulocytes and RDW [9]. This study shows that C15:0 supplementation directly stabilizes red blood cells and reduces the need for the rapid production of new cells. These same results (alleviated anemia with raised hemoglobin and lowered reticulocytes and RDW within 1 to 3 months) occurred among dolphins provided a modified diet higher in C15:0, in which red blood cell membrane C15:0 concentrations were raised from 0.17% to 0.58% total fatty acids [140]. Further, C15:0 supplementation has been shown to lower lipid peroxidation in a mouse model of NAFLD and NASH [50]. These intervention studies are consistent with human studies showing that people with mild anemia have both higher red blood cell membrane C15:0 levels and lower systemic lipid peroxidation compared to people with severe anemia [142].

### 6.2. C15:0 Supplementation Decreases Erythrophagocytosis by Liver Kupffer Cells, Resulting in Attenuated DIOS, NAFLD, and NASH

Liver iron deposition and fibrosis were attenuated within 3 months in a model of anemia, DIOS, and NASH supplemented with C15:0 [47]. This study showed that C15:0 supplementation all but stopped iron deposition in the liver, while also preventing liver fibrosis from advancing from Stage II to Stage III (bridging) fibrosis. Further, a separate mouse model of NAFLD and NASH showed that C15:0 supplementation lowered ALT, AST, liver lipid peroxidation, and liver TNFα and IL-6 [50]. These intervention studies are consistent with human studies showing that people with higher C15:0 concentrations have a lower risk of having NAFLD or severe NASH [73,74].

### 6.3. C15:0 Supplementation Lowered Indices of Insulin Resistance, Metabolic Syndrome, Type 2 Diabetes, and Cardiovascular Disease

C15:0 supplementation lowered cholesterol and triglycerides in two models of metabolic syndrome, type 2 diabetes, and NAFLD [47]. In the type 2 diabetes model, C15:0 supplementation also lowered glucose, IL-6, and MCP-1. These intervention studies are consistent with human studies showing that people with higher C15:0 concentrations have a lower risk of having metabolic syndrome, type 2 diabetes, and cardiovascular disease [43,44,45,59,60,61,62,63,64,65,66,67,68,69,70,71].

In summary, the demonstrated benefits of C15:0 supplementation, including evidence of reversing key stages of the proposed Cellular Fragility Syndrome, support that this syndrome is not only caused by C15:0 deficiencies but can be fixed with C15:0 supplementation.

## 7. Conclusions

In summary, C15:0 is a newly discovered essential fatty acid that has a core role in physically strengthening cell membranes and protecting cells against lipid peroxidation. There is abundant evidence that lower circulating C15:0 concentrations are associated with a higher risk of having type 2 diabetes, cardiovascular disease, and NAFLD. As part of a Cellular Stability Hypothesis, it is proposed that adequate C15:0 concentrations are needed in cell membranes to prevent ferroptosis, a newly discovered method of cell death involving the lipid peroxidation of cell membrane fatty acids and intracellular iron—which has also been linked to type 2 diabetes, cardiovascular disease, and NAFLD.

Based on numerous studies provided in this review, a definition of nutritional C15:0 deficiency is offered (circulating C15:0 ≤ 0.2% total fatty acids). A description of the pathophysiology behind this nutritional C15:0 deficiency syndrome (Cellular Fragility Syndrome) is provided that explains how low C15:0 may accelerate the progression of aging-associated diseases, including dysmetabolic iron overload syndrome (DIOS), type 2 diabetes, cardiovascular disease, and NAFLD. Finally, controlled interventions in relevant models demonstrate that C15:0 supplementation can reverse key components of ferroptosis and Cellular Fragility Syndrome. Historical and recent human data support a daily dietary C15:0 intake of around 100 to 200 mg to achieve circulating C15:0 levels > 20 µM (>5 µg/mL, or >0.2%).

Beyond fixing nutritional deficiencies, there is evidence that optimal circulating C15:0 concentrations (>0.4% to 0.64% total fatty acids) may support long-term cardiovascular health and longevity. Continued studies, including clinical trials, will help further test the Cellular Stability Hypothesis and the proposed definition of nutritional C15:0 deficiencies. Given global declines in dietary C15:0 intake, further studies are needed to better understand the depth and breadth of the proposed C15:0-deficiency-driven Cellular Fragility Syndrome across different human populations and how this syndrome may be contributing to rises in aging-associated diseases, especially among younger people.

## 8. Patents

Patents relevant to this manuscript are available at https://fatty15.com/pages/patents [143,144,145].

## Figures and Tables

**Table 1:** C15:0 content in various fish species ^1^.

Fish Species	C15:0 Content (% Total Fatty Acids)
Mullet	1.18%
Catfish	0.94%
Striped red mullet	0.92%
Sea bass	0.82%
Eel	0.73%
Red porgy	0.63%
Golden grouper	0.62%
Sardine	0.60%
Carp	0.53%
Sole	0.50%
Sea bream	0.42%

^1^ Data extracted from Abouel-Yazeed, 2013 [107].

## Data Availability

Not applicable.
